# Dichotomous mechanisms of aortic stiffening in high‐fat diet fed young and old B6D2F1 mice

**DOI:** 10.1002/phy2.268

**Published:** 2014-03-26

**Authors:** Grant D. Henson, Ashley E. Walker, Kelly D. Reihl, Anthony J. Donato, Lisa A. Lesniewski

**Affiliations:** 1 Department of Exercise and Sports Science, University of Utah, Salt Lake City, Utah; 2 Division of Geriatrics, Department of Internal Medicine, University of Utah, Salt Lake City, Utah; 3 Veteran's Affairs Medical Center‐Salt Lake City, Geriatrics Research Education and Clinical Center, Salt Lake City, Utah

**Keywords:** Advanced glycation end products, arterial stiffness, collagen, elastin, high‐fat diet, pulse wave velocity, structure

## Abstract

Advancing age is associated with increased stiffness of large elastic arteries as assessed by aortic pulse wave velocity (PWV). Greater PWV, associated with increased risk of cardiovascular diseases, may result from altered expression of the extracellular matrix proteins, collagen and elastin, as well as cross‐linking of proteins by advanced glycation end products (AGEs). Indeed, aortic PWV is greater in old (28–31 months) normal chow (NC, 16% fat by kcal)‐fed male B6D2F1 mice compared with young (Y: 5–7 months) NC‐fed mice (397 ± 8 vs. 324 ± 14 cm/s, *P *<**0.05). Aging also induces a ~120% increase in total aortic collagen content assessed by picosirius red stain, a ~40% reduction in medial elastin assessed by Verhoeff's Van Geison stain, as well as a 90% greater abundance of AGEs in the aorta (*P *<**0.05). The typical American diet contains high dietary fat and may contribute to the etiology of arterial stiffening. To that end, we hypothesized that the age‐associated detriments in arterial stiffening are exacerbated in the face of high dietary fat. In young animals, high‐fat (40% fat by kcal) diet increases aortic stiffness by 120 ± 18 cm/s relative to age‐matched NC‐fed mice (*P *<**0.001). High‐fat was without effect on aortic collagen or AGEs content in young animals; however, elastin was greatly reduced (~30%) after high‐fat in young mice. In old animals, high‐fat increased aortic stiffness by 108 ± 47 cm/s but was without effect on total collagen content, medial elastin, or AGEs. These data demonstrate that both aging and high‐fat diet increase aortic stiffness, and although a reduction in medial elastin may underlie increased stiffness in young mice, stiffening of the aorta in old mice after high‐fat diet does not appear to result from a similar structural modification.

## Abstract (precis)

Advancing age and consumption of a high‐fat diet are independently associated with increased stiffness of large elastic arteries as assessed by aortic pulse wave velocity (PWV). We sought to determine if age‐associated detriments in arterial stiffening are exacerbated in the face of high dietary fat. Our findings demonstrate that both aging and high‐fat diet increase aortic stiffness and although a reduction in medial elastin may underlie increased stiffness in young mice, stiffening of the aorta in old mice after HF diet does not appear to result from a similar structural modification.

## Introduction

In industrialized societies, cardiovascular diseases (CVD) are the leading cause of morbidity and mortality (Lakatta and Levy 2003; Redberg et al. 2009). Among CVD, coronary heart disease accounts for nearly half of all deaths (Go et al. 2013). Several factors contribute to the development of cardiovascular disease such as the ingestion of a typical high‐fat diet and advancing age, which is the single greatest, nonmodifiable contributor to CVD risk. The underlying mechanisms by which high‐fat and advancing age contribute to CVD etiology are not fully known, however, increased large artery stiffness has been implicated as a strong predictor of future CVD (Laurent et al. 2005; Sutton‐Tyrrell et al. 2005; Willum Hansen et al. 2006; Go et al. 2013). Arterial stiffness is influenced by acute physiological perturbations such as the consumption of a single high‐fat meal (Mc Clean et al. 2007), as well as chronic physiological stimuli such as stress (Vlachopoulos et al. 2009), long‐term dietary changes (Avolio et al. 1986), and exercise (Vaitkevicius et al. 1993), which, in some cases, lead to permanent or semipermanent structural changes. For example, increased arterial stiffness is linked to greater collagen protein expression with age in the arterial wall (Zieman et al. 2005; Fitch et al. 2006; Diez 2007; Fleenor et al. 2010) and reduced (Fitch et al. 2006) or fragmented elastin in the elastic lamina (Wagenseil et al. 2005). Concurrent with greater collagen expression, arterial stiffening is also associated with increased cross‐linking of collagen fibers (Sims et al. 1996) by protein–glucose interactions termed advanced glycation end products (AGEs; Kass et al. 2001; Wolffenbuttel et al. 1998). These structural modifications together contribute to greater arterial stiffness and thus to elevated risk of CVD morbidity/mortality.

Both obesity and advancing age have been independently linked to greater pulse wave velocity (PWV), an in vivo index of arterial stiffness (Vaitkevicius et al. 1993; Mitchell et al. 2004; Sutton‐Tyrrell et al. 2005; Zebekakis et al. 2005; Safar et al. 2006). However, with an increasing percentage of older adults in the population (The State of Aging and Health in America 2013), the coincidence of older age and consumption of a high‐fat diet is rising (Villareal et al. 2005; Chapman 2008; Go et al. 2013), and these risk factors may interact to compound stiffening of the large arteries and contribute to subsequent CVD morbidity and mortality.

In the present study, we sought to explore the hypothesis that two hallmark CVD risk factors common to the American population, namely advancing age and the chronic consumption of a high‐fat diet, may exert an additive effect on large elastic artery stiffness. To explore this hypothesis, we utilized high‐fat feeding in the B6D2F1 mouse, a strain we have previously demonstrated to develop increased PWV (large artery stiffening) with advanced age (Donato et al. 2013). To explore the additive effect of aging and consumption of high dietary fat, we assessed in vivo arterial stiffness by pulse wave velocity in animals fed either high‐fat (8 weeks, 40% fat by kcal) diet or normal chow (NC) diet and we further sought to elucidate the potential mechanisms contributing to greater stiffness in response to these stimuli by examining changes in key structural components of the artery. Specifically, we hypothesized that advancing age and high‐fat diet would induce altered content of key structural proteins (collagen and elastin) as well as increase the formation of a key modifier of collagen, advanced glycation end products, compared with either condition alone.

## Methods

### Animals

Old B6D2F1 mice were obtained from the National Institute on Aging rodent colony maintained at Charles River Inc. and young mice were obtained from the commercial colony maintained at Charles River Inc. All mice were housed in an animal care facility at the Salt Lake City VA Medical Center's Animal Facility on a 12:12 light:dark cycle. Young (7.6 ± 0.5 months) and old (30.4 ± 0.3 months) male mice were fed normal rodent chow (NC: 16% kcal from fat, 55% carbohydrate, 29% protein, 8640 Harlan Teklad 22/5 Standard Rodent Chow) or a commercially available high‐fat diet (HF: 41% kcal from fat (41% saturated/total fat), 41% from carbohydrate, 18% protein, Harlan Adjusted Fat diet #TD.96132) ad libitum and housed in standard mouse cages for 8–12 weeks prior to sacrifice as previously described (Lesniewski et al. 2013). Access to food and water was provided ad libitum. All animal procedures conformed to the Guide for the Care and Use of Laboratory Animals (2011, 8th Ed) and were approved by the University of Utah and Salt Lake City VA Medical Center Animal Care and Use Committee.

### In vivo pulse wave velocity

The day before euthanasia, aortic PWV was measured as described previously (Hartley et al. 1997; Reddy et al. 2003, 2005, Donato et al. 2013). Briefly, mice were anesthetized under 2% isoflurane in a closed chamber anesthesia machine (V3000PK, Parkland Scientific, Coral Springs, FL) for ~1–3 min. Anesthesia was maintained via nose cone, and mice were secured in a supine position on a heating board (~35°C) to maintain body temperature. Velocities were measured with 4‐mm crystal 20‐MHz Doppler probes (Indus Instruments, Webster, TX) at the transverse aortic arch and ~ 4 cm distal at the abdominal aorta and collected using WinDAQ Pro+ software (DataQ Instruments, Akron, OH). Absolute pulse arrival times were indicated by the sharp upstroke, or foot, of each velocity waveform analyzed with WinDAQ Waveform Browser (DataQ Instruments, Akron, OH). Aortic PWV is then calculated as the quotient of the separation distance, assessed to the nearest half millimeter by engineering caliper (typically ~4 cm) and difference in absolute arrival times.

### Aortic histology

Animals were euthanized via by exsanguination via cardiac puncture under isoflurane anesthesia. Thoracic aortas were quickly excised and placed in cold (4°C) physiological salt solution. Two millimeter rings with perivascular tissue intact were removed from the thoracic aorta directly distal to the greater curvature of the aortic arch and embedded in Optimal Cutting Temperature (OCT) medium. Rings were sectioned (7 micron) and mounted on glass slides for histologic analysis. Collagen was quantified by picrosirius red stain as described previously (Donato et al. 2010) and green channel images from a RGB stack were utilized for densitometric quantification with ImageJ (NIH, Bethesda, MA). Elastin was quantified by Verhoff's Van Geison stain as described previously (Raub et al. 2010) and 8‐bit grayscale were utilized for densitometric quantification with ImageJ. AGEs were assessed by immunohistochemical visualization. Briefly, sections are washed and incubated in primary antibody (1:200, GeneTex 20055) or negative control (2.5% horse serum, Vector Labs) overnight and AGEs were visualized using the appropriate secondary antibody and Vector Labs NovaRed (SK‐4800) Peroxidase substrate kit. Three separate, blinded observers scored images on a zero to three scale (0 = absence of appreciable positive stain, 1 = minimal positive stain, 2 = appreciable positive stain, 3 = highly positive stain). Scores for each section were averaged across observers and normalized to negative control sections.

### Free fatty acid assessment

Plasma nonesterified fatty acid content was measured in blood collected during exsanguination by cardiac puncture using a commercially available kit (Wako Diagnostics, HR‐Series NEFA‐HR).

### Statistics

To detect differences between age and diet groups for pulse wave velocity as well as collagen, elastin, and AGEs content, one‐way analysis of variance were performed with least squares difference post hoc tests utilized where appropriate. All data are represented as mean ± SEM. Significance was set at *P *<**0.05.

## Results

### Animal characteristics

In comparison to young NC‐fed mice, both heart mass and liver mass were greater in old NC mice. Soleus mass, while tending to be greater in aged animals was not significantly different from young counterparts. Although not different with aging in NC‐fed mice, epididymal white adipose tissue (WAT) was higher after HF diet in young and old mice. Serum‐free fatty acid content was not different between groups (Table 1).

**Table 1.** Body, heart, liver, soleus muscle, and epididymal white adipose tissue (WAT) mass and free fatty acids (FFA) in young and old normal chow (NC) and high‐fat (HF)‐fed mice

	Young (Y)		Old (O)	
	NC	HF	NC	HF
*N*	14	13	20	9
Body Mass (g)	32.7 ± 1.34	35.7 ± 0.8	37.2 ± 2.3	39.9 ± 1.7
Heart Mass (g)	0.19 ± 0.01	0.16 ± 0.01	0.26 ± 0.03*	0.20 ± 0.03
Liver Mass (g)	1.87 ± 0.14	1.68 ± 0.06	2.19 ± 0.07*	2.33 ± 0.21
Soleus Mass (g)	0.017 ± 0.001	0.016 ± 0.002	0.023 ± 0.004	0.023 ± 0.009
WAT Mass (g)	0.62 ± 0.05	0.85 ± 0.08*	0.80 ± 0.14	1.25 ± 0.24^†^
Plasma FFA	724 ± 64	843 ± 208	958 ± 195	975 ± 109

Values are mean ± SEM *P <**0.05 versus YC. ^†^*P *<**0.05 versus OC.

#### Pulse wave velocity is markedly increased by age and high‐fat diet alone and in combination

**Figure 1.**
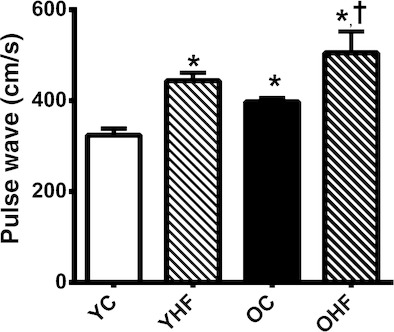
Aortic pulse wave velocity (PWV;* n *=**5–13 per group) in young normal chow (YC) and high‐fat (YHF) fed and old normal chow (OC) and high‐fat (OHF) fed B6D2F1 mice. Values are mean ± SEM. *P <**0.05 versus YC. ^†^*P *<**0.05 versus OC.

When compared with young NC, aortic stiffness, assessed by PWV, was higher in old NC (324 ± 14 vs. 397 ± 8 cm/s, *P *<**0.05) mice. Aortic stiffness was greater in young animals fed HF for 8 weeks (+120 cm/s, *P *<**0.05 compared to young NC) and HF led to a further increase in PWV in old mice (505 ± 47.11 cm/s, *P *<**0.05 compared to both young and old NC; Fig. 1). Heart rates during testing were not significantly different between treatments or ages (data not shown).

#### Aortic collagen content is increased by age but not high‐fat diet

**Figure 2.**
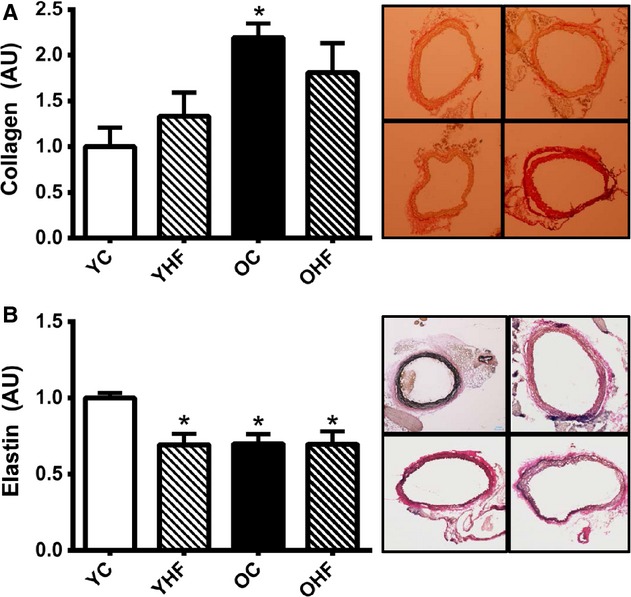
Total thoracic aortic collagen (A) and medial elastin (B) content in young normal chow (YC) and high‐fat (YHF) fed and old normal chow (OC) and high‐fat (OHF)‐fed B6D2F1 mice. Collagen and elastin are expressed normalized to their respective YC means. Representative picrosirius red (collagen) and Verhoeff's Van Geisson (elastin)‐stained aortic rings are provided to the right of the summary graphs (clockwise from upper left: YC, YHF, OC, OHF). Values are mean ± SEM. *P <**0.05 versus YC.

In histological sections of aortas excised from old NC animals, collagen content was 120% higher than young NC (*P *<**0.05). HF did not impact aortic collagen content in young or old mice (Fig. 2A).

#### Aortic elastin content is reduced by aging and high‐fat diet

Aging reduced elastin content in the aortic media by ~30% (*P *<**0.05).The same reduction (~30%) in aortic elastin was observed in young mice after HF (*P *<**0.05); however, there was no further reduction in elastin content in old mice after HF (Fig. 2B).

#### Advanced glycation end products are increased by aging and HF diet in the aorta

**Figure 3.**
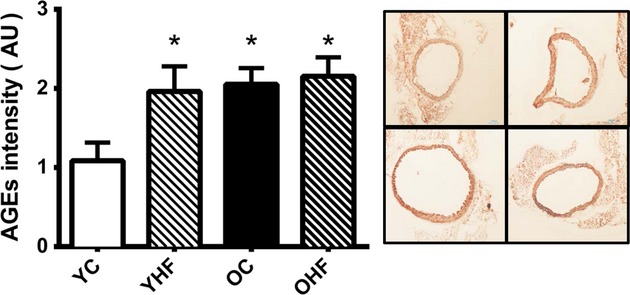
Aortic advanced glycation end products (AGEs) in young normal chow (YC) and high‐fat (YHF) fed and old normal chow (OC) and high‐fat (OHF)‐fed B6D2F1 mice. AGEs identified by immunohistochemistry with primary antibody against AGEs (Genetex) and visualized with NovaRed. Representative stained aortic rings are provided to the right of the summary graphs (clockwise from upper left: YC, YHF, OC, OHF) Values are mean ± SEM *P <**0.05 versus YC.

There was a 90% increase in aortic AGEs of old compared to young NC‐fed mice (*P *<**0.05). This age‐associated increase in AGEs was mirrored in young mice after HF (98% increase, *P *<**0.05). However, the combination of older age and HF did not result in an additive increase in AGEs content (Fig. 3).

## Discussion

The novel findings of the present study are that (i) two major, commonly coincident risk factors for CVD, advancing age and consumption of a high‐fat diet, additively increase arterial stiffness; (ii) high‐fat diet is sufficient to reduce arterial elastin and increase collagen cross‐links by AGEs in young mice; and (iii) while advancing age and high‐fat diet induce structural abnormalities in the arterial wall independently, these structural modifications are not the main contributor to the additive effect of older age and high‐fat diet on arterial stiffening.

### Large elastic artery stiffening and structural alterations with aging

The results of the present study are in agreement with previously published reports that demonstrated an age‐associated increase in large artery stiffness with aging, assessed by aortic pulse wave velocity in humans (McEniery et al. 2005) and mice (Donato et al. 2013), and a decreased distensibility in isolated carotid arteries from old mice (Donato et al. 2013). Stiffening of the carotid and aorta, large conduit arteries, was associated with structural modifications in the arterial wall (Donato et al. 2013). Here, we demonstrate that concurrent with an increase in aortic stiffness assessed in vivo, there is an increase in collagen deposition throughout the arterial wall, as well as reduced elastin content in the elastic lamina, similar to what has been found previously in aged humans (Goldin et al. 2006) and rodents (Donato et al. 2010). Importantly, and in agreement with others (Fleenor et al. 2012; LaRocca et al. 2013), we also found an age‐associated increase in advanced glycation end products. These data are significant given the efficacy of AGEs breakers in treating diabetic aortic stiffness in rats (Wolffenbuttel et al. 1998). Indeed, clinical treatment with ALT‐711, an advanced glycation end product breaker, has been shown to increase arterial compliance, reducing pulse wave velocity in a hypertensive patient population (Kass et al. 2001).

### Contribution of high‐fat diet to arterial structure

We have previously demonstrated that high‐fat diet is associated with reduced arterial function in the B6D2F1 mouse (Lesniewski et al. 2012). It is also appreciated that obesity, and even acute ingestion of a single high‐fat meal (Lithander et al. 2013), induce alterations in in vivo arterial stiffness. However, there are limited data exploring the contribution of a high‐fat diet per se to arterial structural components. Data from obesity‐related studies suggest that obesity is strongly associated with increased arterial stiffness (Sutton‐Tyrrell et al. 2001; Wildman et al. 2003), and it is well‐appreciated that chronic exposure to high‐fat diet induces obesity in humans and animals. While our animals did not gain weight over the treatment period, our data are the first to demonstrate that high‐fat diet per se*,* even independent of obesity, induces structural maladaptations in the arterial wall; notably, reduced elastin content of the medial layer. Elastin can be degraded by elastases such as matrix metalloproteinase (MMP)‐9 (Lau et al. 2008). Importantly, MMP‐9 has been shown to be elevated in serum from obese patients (Ghanim et al. 2004) and in normal weight subjects following ingestion of a single high‐fat, high‐carbohydrate meal (Ghanim et al. 2010), providing a potential mechanism for the changes in elastin observed in this study. We further show that high‐fat diet is sufficient to induce a greater expression of AGEs in the aorta of young animals. Previous studies have found similar increases in AGEs high‐fat in the viscera of high‐fat fed animals (Li et al. 2005) or in the arteries of diabetic mice (Reaven et al. 1997), but, to our knowledge, we are the first to demonstrate the high‐fat diet increases AGEs in the arterial wall.

### Combined effect of aging and diet on large elastic arteries

Chronic consumption of a typical American high‐fat diet can lead to obesity as well as a variety of pathophysiological consequences such as hepatic steatosis (Ma et al. 2008), diabetes (Ravussin and Smith 2002; Lesniewski et al. 2007) and vascular endothelial dysfunction (Lesniewski et al. 2008; Donato et al. 2012). The latter may result from, or contribute to, increased arterial stiffness. In humans, few studies have explored the combined effects of obesity and aging on arterial stiffening, and the few available reports have contradictory results. While a report by Corden et al. (2013) found that obesity is associated with greater pulse wave velocity in older adults and lower pulse wave velocity (PWV) in children, Wildman et al. (2003) demonstrated that obesity is associated with a 40 to 90 cm/s increase in pulse wave velocity across age groups. Similar to the results obtained in obese animals by Wildman et al., we demonstrate here that consumption of high‐fat diet per se increased pulse wave velocity in both young and old mice and that the increase was similar in magnitude (~100 cm/s) in both age groups. However, the important distinction is that our data suggest that high‐fat diet, even in the absence of overt obesity, induces adverse physiological consequences such as arterial stiffness.

Furthermore, we extended these findings to explore the potential changes in arterial structural components that may underlie alterations in arterial stiffness. Our data provide evidence for differential mechanisms contributing to increased arterial stiffness after high‐fat diet between young and old mice. Nevertheless, given the higher initial pulse wave velocity in old normal show fed mice, the increased pulse wave velocity after high‐fat diet in old mice results in a large increase in arterial stiffness that may be a critical factor in the increased risk of disease and mortality when aging is combined with high‐fat diet. These results are highly significant given that the population of older adults in the United States is expected to double by 2050 (Unal et al. 2010) and that obesity has increased steadily in this age group from 1999 to 2010 (Fakhouri et al. 2012). Consequently, a recent report demonstrates that the lifetime health care costs are significantly increased and health outcomes are significantly worsened by overweight/obesity in Americans above age 65 (Yang and Hall 2008).

### Dichotomous structural modifications in young and old mice

We have previously demonstrated that aging is related to key structural changes within the arterial wall typically associated with greater stiffness (Donato et al. 2013). Namely, the deposition of collagen concomitant with reduced or fragmented elastin and greater formation of advanced glycation end products (Donato et al. 2013). Thus, we hypothesized that an additive effect of high‐fat diet and aging would be reflected in arterial structural changes. However, although we did observe an increase in pulse wave velocity in old high‐fat diet fed mice compared with diet‐ and age‐control animals, we did not observe the same structural modifications in old high‐fat fed mice as were observed in young mice after high‐fat diet. One possible explanation for the dichotomy of changes in the structural composition arteries is that aging or chronic high‐fat diet in youth is associated with significant structural changes; however, in older age, the established modifications in the arterial wall are at or near the physiological threshold. Dysfunction of the arterial endothelium, a well‐established phenomenon that occurs with advancing age and after high‐fat diet, both independently and in combination, may underlie the additive effect of aging and high‐fat diet on PWV (Lesniewski et al. 2008; Donato et al. 2012, 2013). Endothelial dysfunction is characterized by a blunted vasodilatory response to pharmacological stimulation in ex vivo vessel preparations. In young mice fed high‐fat diet, impaired endothelium‐dependent dilation was associated with a greater mechanical stiffness of the carotid artery, calculated from the passive mechanical properties in excised carotid artery. However, although consumption of a high‐fat diet also reduced endothelium‐dependent dilation to acetylcholine in old mice compared to age‐matched normal chow fed mice, this was not associated with greater mechanical stiffness (Lesniewski et al. 2013). We further demonstrated that the old animals fed high‐fat diet had a reduced nitric oxide bioavailability compared to age‐matched normal chow fed counterparts that was abolished by superoxide scavenging. Taken together with the present findings demonstrating a lack of change in structural properties in the old high‐fat fed mice, these previous results implicate an oxidative stress‐mediated reduction in endothelial function in increased in vivo arterial stiffness after high‐fat diet in old mice. Indeed, both reductions in nitric oxide (Fitch et al. 2001) and greater superoxide (Delles et al. 2008; Donato et al. 2013) have been related to increases in arterial stiffness. This provides evidence that additional factor(s), perhaps related to vascular cell signaling, may confer stiffness to the artery in addition to structural or mechanical alterations.

## Conclusions and Implications

Our data demonstrate that there is an effect of both age and high‐fat diet on aortic pulse wave velocity, a major predictor of future CVD‐related morbidities and mortalities. Here, we provide evidence that in young animals fed high‐fat diet and old animals fed normal chow, increased pulse wave velocity is associated with greater collagen, reduced elastin, and greater advanced glycation end products than their young normal chow fed counterparts. Furthermore, although old animals fed a high‐fat diet had greater pulse wave velocity than normal chow fed age‐matched mice, this increase in large artery stiffness did not result from further alterations in structural proteins; perhaps indicating a role of endothelial dysfunction in the large artery stiffening observed in these mice.

## Limitations

We acknowledge that there are several limitations to the current study. In vivo pulse wave velocity is a powerful tool for the assessment of arterial stiffening. It is strongly associated with blood pressure and does not allow for mechanistic dissection of the observed changes in structural proteins. Unfortunately, our study lacks blood pressure measures that would allow us to evaluate if the relation between blood pressure and PWV is impacted by high‐fat diet in old mice. Additionally, while the absolute change in structural proteins such as collagen and elastin provide some mechanistic insights into alterations in arterial stiffness, we are not able to assess quantitative changes in the architecture of these proteins. For example, changes in the structure of collagen fibers and the fragmentation of elastin in the elastic lamina may contribute as much as, if not more than, total protein content. Future studies should also determine if the observed structural changes are preventable and/or reversible by pharmacological (e.g., protein crosslink breakers or beta‐adrenergic blockade) or lifestyle (e.g., chronic aerobic exercise or intermittent fasting) interventions, providing valuable links between structural and functional outcomes in arteries.

## Summary

The novel findings of the present study demonstrate that both aging and high‐fat diet increase aortic stiffness. We further find that although a reduction in medial elastin may underlie increased stiffness in young mice, stiffening of the aorta in old mice after high‐fat diet does not appear to result from a similar structural modification. The results of these studies suggest that the underlying mechanisms for high‐fat diet‐induced large artery stiffening differ with age, and thus effective therapies to ameliorate arterial stiffness may need to be targeted to specific populations; that is, therapies that are effective in young populations may not be efficacious in older individuals.

## Acknowledgments

All experiments were performed in the Translational Vascular Physiology Laboratory at the University of Utah and VAMC‐SLC GRECC.

## Conflicts of Interest

None declared.

## References

[b1] Avolio A. P. Clyde K. M. Beard T. C. Cooke H. M. Ho K. O'Rourke M. F. 1986 Improved arterial distensibility in normotensive subjects on a low salt diet. Arterioscler. Thromb. Vasc. Biol.; 6:166-169. 10.1161/01.atv.6.2.166 3954670

[b2] Chapman I. M. 2008 Obesity in old age. Front. Horm. Res.; 36:97-106. 18230897 10.1159/000115358

[b3] Corden B. Keenan N. G. de Marvao A. S. Dawes T. J. DeCesare A. Diamond T. 2013 Body fat is associated with reduced aortic stiffness until middle age. Hypertension; 61:1322-1327. 23608657 10.1161/HYPERTENSIONAHA.113.01177

[b4] Delles C. Zimmerli L. U. McGrane D. J. Koh‐Tan C. H. Pathi V. L. McKay A. J. 2008 Vascular stiffness is related to superoxide generation in the vessel wall. J. Hypertens.; 26:946-955. 18398337 10.1097/HJH.0b013e3282f7677c

[b5] Diez J. 2007 Arterial stiffness and extracellular matrix. Adv. Cardiol.; 44:76-95. 17075200 10.1159/000096722

[b6] Donato A. J. Henson G. D. Morgan R. G. Enz R. A. Walker A. E. Lesniewski L. A. 2012 TNF‐*α* impairs endothelial function in adipose tissue resistance arteries of mice with diet‐induced obesity. Am. J. Physiol. Heart Circ. Physiol.; 303:H672-H679. 22821989 10.1152/ajpheart.00271.2012 PMC3468456

[b7] Donato A. J. Walker A. E. Magerko K. Bramwell R. C. Black A. D. Henson G. D. 2013 Life‐long caloric restriction reduces oxidative stress and preserves nitric oxide bioavailability and function in arteries of old mice. Aging Cell; 12:772-783. 23714110 10.1111/acel.12103 PMC3772986

[b8] Fakhouri T. H. Ogden C. L. Carroll M. D. Kit B. K. Flegal K. M. 2012 Prevalence of obesity among older adults in the United States 2007‐2010. NCHS Data Brief; 106:1-8. 23102091

[b9] Fitch R. M. Vergona R. Sullivan M. E. Wang Y.‐X. 2001 Nitric oxide synthase inhibition increases aortic stiffness measured by pulse wave velocity in rats. Cardiovasc. Res.; 51:351-358. 11470475 10.1016/s0008-6363(01)00299-1

[b10] Fitch R. M. Rutledge J. C. Wang Y.‐X. Powers A. F. Tseng J.‐L. Clary T. 2006 Synergistic effect of angiotensin II and nitric oxide synthase inhibitor in increasing aortic stiffness in mice. Am. J. Physiol. Heart Circ. Physiol.; 290:H1190-H1198. 16272204 10.1152/ajpheart.00327.2005

[b11] Fleenor B. S. Marshall K. D. Durrant J. R. Lesniewski L. A. Seals D. R. 2010 Arterial stiffening with ageing is associated with transforming growth factor‐beta1‐related changes in adventitial collagen: reversal by aerobic exercise. J. Physiol.; 588:3971-3982. 20807791 10.1113/jphysiol.2010.194753 PMC3000586

[b12] Fleenor B. S. Sindler A. L. Eng J. S. Nair D. P. Dodson R. B. Seals D. R. 2012 Sodium nitrite de‐stiffening of large elastic arteries with aging: role of normalization of advanced glycation end‐products. Exp. Gerontol.; 47:588-594. 22588062 10.1016/j.exger.2012.05.004 PMC3389216

[b13] Ghanim H. Aljada A. Hofmeyer D. Syed T. Mohanty P. Dandona P. 2004 Circulating mononuclear cells in the obese are in a proinflammatory state. Circulation; 110:1564-1571. 15364812 10.1161/01.CIR.0000142055.53122.FA

[b14] Ghanim H. Sia C. L. Upadhyay M. Korzeniewski K. Viswanathan P. Abuaysheh S. 2010 Orange juice neutralizes the proinflammatory effect of a high‐fat, high‐carbohydrate meal and prevents endotoxin increase and Toll‐like receptor expression. Am. J. Clin. Nutr.; 91:940-949. 20200256 10.3945/ajcn.2009.28584 PMC2844681

[b15] Go A. S. Mozaffarian D. Roger V. L. Benjamin E. J. Berry J. D. Borden W. B. 2013 Heart disease and stroke statistics—2013 update: a report from the american heart association. Circulation; 127:e6-e245. 23239837 10.1161/CIR.0b013e31828124ad PMC5408511

[b16] Goldin A. Beckman J. A. Schmidt A. M. Creager M. A. 2006 Advanced glycation end products: sparking the development of diabetic vascular injury. Circulation; 114:597-605. 16894049 10.1161/CIRCULATIONAHA.106.621854

[b117] Guide for the Care and Use of Laboratory Animals. 2011 National Academies Washington, D. C‐‐‐‐ 21595115

[b17] Hartley C. J. Taffet G. E. Michael L. H. Pham T. T. Entman M. L. 1997 Noninvasive determination of pulse‐wave velocity in mice. Am. J. Physiol.; 273:H494-H500. 9249523 10.1152/ajpheart.1997.273.1.H494

[b18] Kass D. A. Shapiro E. P. Kawaguchi M. Capriotti A. R. Scuteri A. deGroof R. C. 2001 Improved arterial compliance by a novel advanced glycation end‐product crosslink breaker. Circulation; 104:1464-1470. 11571237 10.1161/hc3801.097806

[b19] Lakatta E. G. Levy D. 2003 Arterial and cardiac aging: major shareholders in cardiovascular disease enterprises: Part I: aging arteries: a “set up” for vascular disease. Circulation; 107:139-146. 12515756 10.1161/01.cir.0000048892.83521.58

[b20] LaRocca T. J. Gioscia‐Ryan R. A. Hearon C. M. Jr Seals D. R. 2013 The autophagy enhancer spermidine reverses arterial aging. Mech. Ageing Dev.; 134:314-320. 23612189 10.1016/j.mad.2013.04.004 PMC3700669

[b21] Lau A. C. Duong T. T. Ito S. Yeung R. S. 2008 Matrix metalloproteinase 9 activity leads to elastin breakdown in an animal model of Kawasaki disease. Arthritis Rheum.; 58:854-863. 18311803 10.1002/art.23225

[b22] Laurent S. Boutouyrie P. Lacolley P. 2005 Structural and genetic bases of arterial stiffness. Hypertension; 45:1050-1055. 15851625 10.1161/01.HYP.0000164580.39991.3d

[b23] Lesniewski L. A. Hosch S. E. Neels J. G. de Luca C. Pashmforoush M. Lumeng C. N. 2007 Bone marrow–specific Cap gene deletion protects against high‐fat diet–induced insulin resistance. Nat. Med.; 13:455-462. 17351624 10.1038/nm1550

[b24] Lesniewski L. A. Donato A. J. Behnke B. J. Woodman C. R. Laughlin M. H. Ray C. A. 2008 Decreased NO signaling leads to enhanced vasoconstrictor responsiveness in skeletal muscle arterioles of the ZDF rat prior to overt diabetes and hypertension. Am. J. Physiol. Heart Circ. Physiol.; 294:H1840-H1850. 18245568 10.1152/ajpheart.00692.2007 PMC2646849

[b25] Lesniewski L. A. Zigler M. L. Durrant J. R. Nowlan M. J. Folian B. J. Donato A. J. 2013 Aging compounds western diet‐associated large artery endothelial dysfunction in mice: prevention by voluntary aerobic exercise. Exp. Gerontol.; 48:1218-1225. 23954368 10.1016/j.exger.2013.08.001 PMC3840721

[b26] Li S. Y. Liu Y. Sigmon V. K. McCort A. Ren J. 2005 High‐fat diet enhances visceral advanced glycation end products, nuclear O‐Glc‐Nac modification, p38 mitogen‐activated protein kinase activation and apoptosis. Diabetes Obes. Metab.; 7:448-454. 15955132 10.1111/j.1463-1326.2004.00387.x

[b27] Lithander F. E. Herlihy L. K. Walsh D. M. Burke E. Crowley V. Mahmud A. 2013 Postprandial effect of dietary fat quantity and quality on arterial stiffness and wave reflection: a randomised controlled trial. Nutr. J.; 12:93 23841960 10.1186/1475-2891-12-93 PMC3717051

[b28] Ma X. Hua J. Li Z. 2008 Probiotics improve high fat diet‐induced hepatic steatosis and insulin resistance by increasing hepatic NKT cells. J. Hepatol.; 49:821-830. 18674841 10.1016/j.jhep.2008.05.025 PMC2588670

[b29] Mc Clean C. Mc Laughlin J. Burke G. Murphy M. Trinick T. Duly E. 2007 The effect of acute aerobic exercise on pulse wave velocity and oxidative stress following postprandial hypertriglyceridemia in healthy men. Eur. J. Appl. Physiol.; 100:225-234. 17323071 10.1007/s00421-007-0422-y

[b30] McEniery C. M. Yasmin X. Hall I. R. Qasem A. Wilkinson I. B. Cockcroft J. R. 2005 Normal Vascular aging: differential effects on wave reflection and aortic pulse wave velocity: the Anglo‐Cardiff Collaborative Trial (ACCT). J. Am. Coll. Cardiol.; 46:1753-1760. 16256881 10.1016/j.jacc.2005.07.037

[b31] Mitchell G. F. Parise H. Benjamin E. J. Larson M. G. Keyes M. J. Vita J. A. 2004 Changes in arterial stiffness and wave reflection with advancing age in healthy men and women the Framingham Heart Study. Hypertension; 43:1239-1245. 15123572 10.1161/01.HYP.0000128420.01881.aa

[b33] Raub C. B. Mahon S. Narula N. Tromberg B. J. Brenner M. George S. C. 2010 Linking optics and mechanics in an in vivo model of airway fibrosis and epithelial injury. J. Biomed. Opt.; 15:015004‐1–015004‐9 20210444 10.1117/1.3322296 PMC2844131

[b34] Ravussin E. Smith S. R. 2002 Increased fat intake, impaired fat oxidation, and failure of fat cell proliferation result in ectopic fat storage, insulin resistance, and type 2 diabetes mellitus. Ann. N. Y. Acad. Sci.; 967:363-378. 12079864 10.1111/j.1749-6632.2002.tb04292.x

[b35] Reaven P. Merat S. Casanada F. Sutphin M. Palinski W. 1997 Effect of streptozotocin‐induced hyperglycemia on lipid profiles, formation of advanced glycation endproducts in lesions, and extent of atherosclerosis in LDL receptor‐deficient mice. Arterioscler. Thromb. Vasc. Biol.; 17:2250-2256. 9351397 10.1161/01.atv.17.10.2250

[b36] Redberg R. F. Benjamin E. J. Bittner V. Braun L. T. Goff D. C. Jr Havas S. 2009 AHA/ACCF [corrected] 2009 performance measures for primary prevention of cardiovascular disease in adults: a report of the American College of Cardiology Foundation/American Heart Association task force on performance measures (writing committee to develop performance measures for primary prevention of cardiovascular disease): developed in collaboration with the American Academy of Family Physicians; American Association of Cardiovascular and Pulmonary Rehabilitation; and Preventive Cardiovascular Nurses Association: endorsed by the American College of Preventive Medicine, American College of Sports Medicine, and Society for Women's Health Research. Circulation; 120:1296-1336. 19770388 10.1161/CIRCULATIONAHA.109.192617

[b37] Reddy A. K. Li Y. H. Pham T. T. Ochoa L. N. Trevino M. T. Hartley C. J. 2003 Measurement of aortic input impedance in mice: effects of age on aortic stiffness. Am. J. Physiol. Heart Circ. Physiol.; 285:H1464-H1470. 12775560 10.1152/ajpheart.00004.2003

[b38] Reddy A. K. Jones A. D. Martono C. Caro W. A. Madala S. Hartley C. J. 2005 Pulsed Doppler signal processing for use in mice: design and evaluation. IEEE Trans. Biomed. Eng.; 52:1764-1770. 16235662 10.1109/tbme.2005.855710

[b39] Safar M. E. Czernichow S. Blacher J. 2006 Obesity, arterial stiffness, and cardiovascular risk. J. Am. Soc. Nephrol.; 17:S109-S111. 16565231 10.1681/ASN.2005121321

[b40] Sims T. Rasmussen L. Oxlund H. Bailey A. 1996 The role of glycation cross‐links in diabetic vascular stiffening. Diabetologia; 39:946-951. 8858217 10.1007/BF00403914

[b41] Sutton‐Tyrrell K. Newman A. Simonsick E. M. Havlik R. Pahor M. Lakatta E. 2001 Aortic stiffness is associated with visceral adiposity in older adults enrolled in the study of health, aging, and body composition. Hypertension; 38:429-433. 11566917 10.1161/01.hyp.38.3.429

[b42] Sutton‐Tyrrell K. Najjar S. S. Boudreau R. M. Venkitachalam L. Kupelian V. Simonsick E. M. 2005 Elevated aortic pulse wave velocity, a marker of arterial stiffness, predicts cardiovascular events in well‐functioning older adults. Circulation; 111:3384-3390. 15967850 10.1161/CIRCULATIONAHA.104.483628

[b32] The State of Aging and Health in America. 2013 Centers for Disease Control and Prevention Atlanta, GA US Dept of Health and Human Services

[b43] Unal R. Yao‐Borengasser A. Varma V. Rasouli N. Labbate C. Kern P. A. 2010 Matrix metalloproteinase‐9 is increased in obese subjects and decreases in response to pioglitazone. J. Clin. Endocrinol. Metab.; 95:2993-3001. 20392866 10.1210/jc.2009-2623 PMC2902064

[b44] Vaitkevicius P. V. Fleg J. L. Engel J. H. O'Connor F. C. Wright J. G. Lakatta L. E. 1993 Effects of age and aerobic capacity on arterial stiffness in healthy adults. Circulation; 88:1456-1462. 8403292 10.1161/01.cir.88.4.1456

[b45] Villareal D. T. Apovian C. M. Kushner R. F. Klein S. 2005 Obesity in older adults: technical review and position statement of the American Society for Nutrition and NAASO, The Obesity Society. Am. J. Clin. Nutr.; 82:923-934. 16280421 10.1093/ajcn/82.5.923

[b46] Vlachopoulos C. Xaplanteris P. Alexopoulos N. Aznaouridis K. Vasiliadou C. Baou K. 2009 Divergent effects of laughter and mental stress on arterial stiffness and central hemodynamics. Psychosom. Med.; 71:446-453. 19251872 10.1097/PSY.0b013e318198dcd4

[b47] Wagenseil J. E. Nerurkar N. L. Knutsen R. H. Okamoto R. J. Li D. Y. Mecham R. P. 2005 Effects of elastin haploinsufficiency on the mechanical behavior of mouse arteries. Am. J. Physiol. Heart Circ. Physiol.; 289:H1209-H1217. 15863465 10.1152/ajpheart.00046.2005

[b48] Wildman R. P. Mackey R. H. Bostom A. Thompson T. Sutton‐Tyrrell K. 2003 Measures of obesity are associated with vascular stiffness in young and older adults. Hypertension; 42:468-473. 12953016 10.1161/01.HYP.0000090360.78539.CD

[b49] Willum Hansen T. Staessen J. A. Torp‐Pedersen C. Rasmussen S. Thijs L. Ibsen H. 2006 Prognostic value of aortic pulse wave velocity as index of arterial stiffness in the general population. Circulation; 113:664-670. 16461839 10.1161/CIRCULATIONAHA.105.579342

[b50] Wolffenbuttel B. H. R. Boulanger C. M. Crijns F. R. L. Huijberts M. S. P. Poitevin P. Swennen G. N. M. 1998 Breakers of advanced glycation end products restore large artery properties in experimental diabetes. Proc. Natl Acad. Sci.; 95:4630-4634. 9539789 10.1073/pnas.95.8.4630 PMC22541

[b51] Yang Z. Hall A. G. 2008 The financial burden of overweight and obesity among elderly Americans: the dynamics of weight, longevity, and health care cost. Health Serv. Res.; 43:849-868. 18454771 10.1111/j.1475-6773.2007.00801.x PMC2442233

[b52] Zebekakis P. E. Nawrot T. Thijs L. Balkestein E. J. van der Heijden‐Spek J. Van Bortel L. M. 2005 Obesity is associated with increased arterial stiffness from adolescence until old age. J. Hypertens.; 23:1839-1846. 16148607 10.1097/01.hjh.0000179511.93889.e9

[b53] Zieman S. J. Melenovsky V. Kass D. A. 2005 Mechanisms, pathophysiology, and therapy of arterial stiffness. Arterioscler. Thromb. Vasc. Biol.; 25:932-943. 15731494 10.1161/01.ATV.0000160548.78317.29

