# Corrigendum

**DOI:** 10.14814/phy2.12062

**Published:** 2014-07-22

## Introduction

Dichotomous mechanisms of aortic stiffening in high‐fat diet‐fed young and old B6D2F1 mice

Grant D. Henson, Ashley E. Walker, Kelly D. Reihl, Anthony J. Donato and Lisa A. Lesniewski

*Physiol Rep*, 2 (3), 2014, e00268, doi: 10.1002/phy2.268

The authors would like to add the following to the Acknowledgments section:

We would like to acknowledge Sugata Hazra for her assistance with the plasma‐free fatty acid measurements.

